# Supplementary material for: Polyhydride CeH9 with an atomic-like hydrogen clathrate structure
Source: Nat Commun. 2019 Aug 1;10:3461. doi: 10.1038/s41467-019-11330-6 (PMC6671988; doi:10.1038/s41467-019-11330-6)
Supplement: Supplementary file 1 — Supplementary Information [file 41467_2019_11330_MOESM1_ESM.pdf]

Supplementary Materials

of

Polyhydride CeH_9 with an atomic-like hydrogen clathrate structure

Xin Li[1], Xiaoli Huang[1]*, Defang Duan[1,2], Chris J. Pickard[2], Di zhou[1], Hui Xie[1], Quan Zhuang[1], Yanping Huang[1], Qiang Zhou[1], Bingbing Liu[1] and Tian Cui[1]*

[1]State Key Laboratory of Superhard Materials, College of Physics, Jilin University, Changchun 130012, China.

[2]Department of Materials Science & Metallurgy, University of Cambridge, 27 Charles Babbage Road, Cambridge CB3 0FS, United Kingdom.

*Corresponding author: huangxiaoli@jlu.edu.cn and cuitian@jlu.edu.cn

Determination of stoichiometries of Ce polyhydrides.

We have determined the stoichiometries and crystal structures of new phases in three aspects in main paper: 1) X-ray diffraction data give the possible phase transitions and symmetry and match the structures of prediction; 2) The volume changes obtained from XRD data indicate the stoichiometry of new phases; 3) Theoretical calculations help us to finally determine the crystal structure and stoichiometry through the calculated enthalpy difference, pressure-volume relation and phase stability. Particularly, the Rietveld refinement combines the calculated and experimental XRD results. We would like to expound detailed distinction of CeH_3, CeH_{3+x} and CeH_4, and $CeH_{9-\delta}$ and CeH_9.

The determination of CeH_3-*Fm*-3*m*

In Supplementary Fig. 3, we found that the patterns of fcc phase match the known structures of CeH_3 and CeH_2. Both CeH_3 and CeH_2 have the same space group *Fm*-3*m*, but CeH_3 has one more hydrogen atom than CeH_2 at interstitial sites 4*b* (0,0,1/2). They have very similar lattice parameters and compression *P*-*V* curves under pressure. Here we propose fcc phase as CeH_3 rather than CeH_2, because the H_2 is excessive in the chamber and the enthalpy calculations indicate that CeH_3 has a lower enthalpy value than CeH_2 (Supplementary Fig. 5).

The stoichiometries of CeH_{3+x}.

From 40 GPa to 72 GPa, Rietveld structural refinement and calculated enthalpy difference (Fig. 1b and Supplementary Fig. 5) prefer the structure of CeH_3-*Pm*-3*n*. Because of the low atomic scattering power of hydrogen, we couldn't determine the content of hydrogen directly through XRD data. In fact, the hydrogen content of cerium hydrides was changing with increasing pressure in the experiment. In order to avoid the confusion and guarantee the accuracy, here we defined the second cubic phase as CeH_{3+x} ($0.7 \leq x \leq 1.1$) and the reasons are as follows. As seen in Supplementary Fig. 6, the volume of CeH_{3+x} is close to CeH_3-*Fm*-3*m* at 40 GPa, however the volume becomes close to CeH_4-*I*4/*mmm* at 72 GPa. In this pressure range, we can also intuitively see that the compression curve of CeH_{3+x} is approaching the mixed line of Ce+2H_2 and connects the curves of CeH_3-*Fm*-3*m* and CeH_4-*I*4/*mmm* smoothly, which also indicates a process of hydrogen absorption. At 40 GPa, $V(CeH_{3+x})$ = 30.54 Å^3/f.u.; V(Ce) = 17.98 Å^3/f.u. and V(H) = 3.4 Å^3/f.u. at this pressure. Thus the stoichiometry of cerium hydrides CeH_{3+x} is x=0.7. At 72 GPa, $V(CeH_{3+x})$ is 27.21 Å^3/f.u.; V(Ce)

16.01 Å^3/f.u. and V(H) is 2.7 Å^3/f.u., which gives x=1.1.

The distinguish of $CeH_{9-\delta}$ and CeH_9.

For CeH_9: We determine the phase of CeH_9-$P6_3/mmc$ through Rietveld refinement of experimental XRD data (Fig. 1b), good agreement between experimental and calculated P-V relation (Fig. 2) and advantage of calculated enthalpy (Supplementary Fig. 5).

For $CeH_{9-\delta}$: Our XRD data match the predicted CeH_8-$P6_3mc$. We exclude this structure because of the imaginary vibration modes in phonon dispersion curves. CeH_9-$P6_3/mmc$ also matches XRD pattern profile, but we distinguish them by the P-V relationship (Fig. 2). We have determined the phase of CeH_9-$P6_3/mmc$ above 103 GPa, however, in the pressure range of 84-103 GPa, we observe abnormal movement of peaks from $CeH_{9-\delta}$ (Supplementary Fig. 13) which indicates the increase of lattice parameters, i.e. the volume expansion of unit cell. Continuously significant expansion of volume is attributed to absorption of hydrogen (Fig. 2 and Supplementary Fig. 13), indicating a gradual hydrogenation process of forming CeH_9, thus we assign the phase in this pressure range noted as $CeH_{9-\delta}$. At 88 GPa, the volumes of Ce and CeH_9 (extrapolation through BM equation) are V(Ce) = 15.37 Å^3/f.u. and $V(CeH_9)$ = 33.91 Å^3/f.u., then the estimation of hydrogen expansion is V(H) = 2.06 Å^3/atom. Therefore, $V(CeH_{9-\delta})$ of 32.17 Å^3/f.u. gives the value range $\delta \leq 0.85$.

Calculation

Here we have used DFT calculations with GGA+U to obtain the lattice parameters and cell volume at different pressures. As reported in previous literatures, Ce atoms with *f* electrons are an extremely delicate system for DFT calculations with the use of standard approximations. We have carefully checked the calculated data, and found that the used GGA+U reproduced the experimental data well and the volume difference between experimental and calculated data is below 3% (Supplementary Fig. 11). In this regard, the current theoretical GGA+U approximations give the correct EOS, which is in agreement with the experimental one. Besides, the GGA+U calculations have been done on band structure, DOS, ELF and phonon spectrum.

It is reported that the anharmonic effects maybe affect the vibrational frequencies, thermodynamics and lattice stability of superhydrides. Errea *et al*. have found that the quantum nature of the proton fundamentally changes the superconducting phase diagram of H_3S. But among these properties, the current work is mainly involved with the calculated phase stability or phase diagram. By comparing with the theoretical and experimental phase stability (Supplementary Fig. 5 and Supplementary Fig. 14), we can find that the range of corresponding phase stability is slightly different with the transition pressure, which maybe contributed by the anharmonic effects. Compared with high symmetry *Im*-3*m* of H_3S, the lower symmetry $P6_3/mmc$ CeH_9 makes the calculation with anharmonic effect difficult. The calculation with anharmonic effects is also extremely hard for the compounds with multi-atoms such as ten-atoms CeH_9. A more delicate calculation is further required for theorist in the future study.

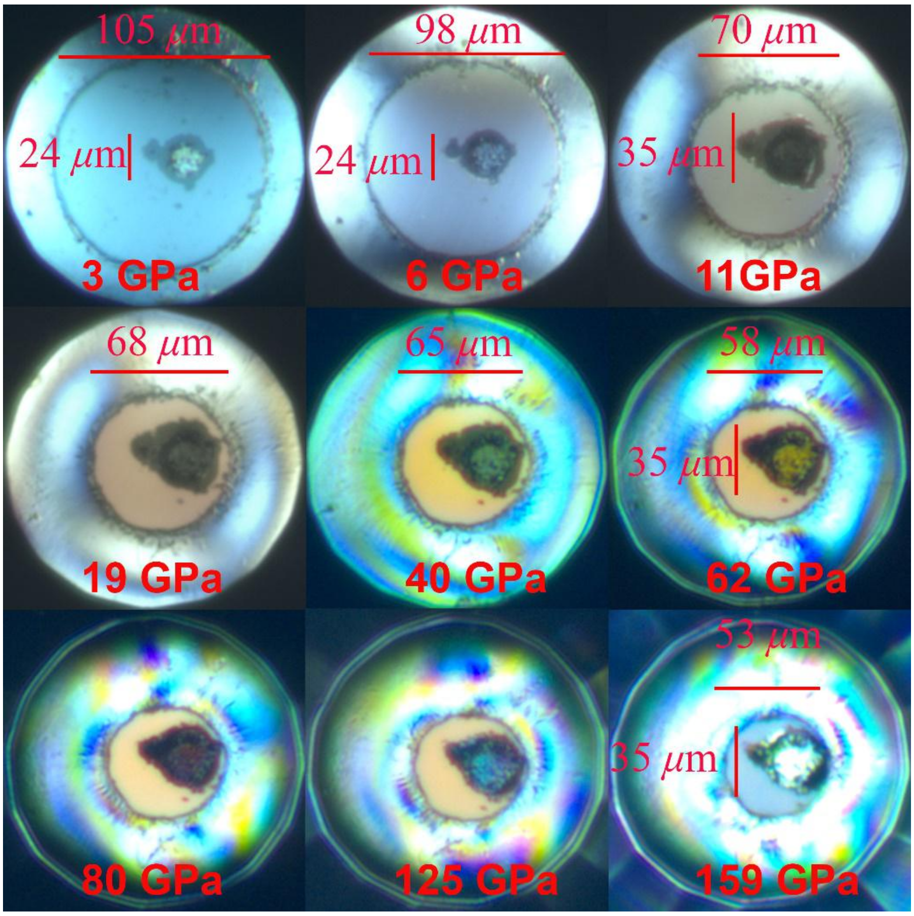

Supplementary Fig. 1 | The microphotoes of the sample chamber at selected pressure. After loading hydrogen, the sealed pressure is 3 GPa with about 24 μm Ce sample. With increasing pressure, the volume of black solid sample increases about 46% accompanied with the decrease of hydrogen sample. Above 34 GPa, the sample chamber remains unchanged, and the excess solid hydrogen remained transparent up to the highest pressure 159 GPa.

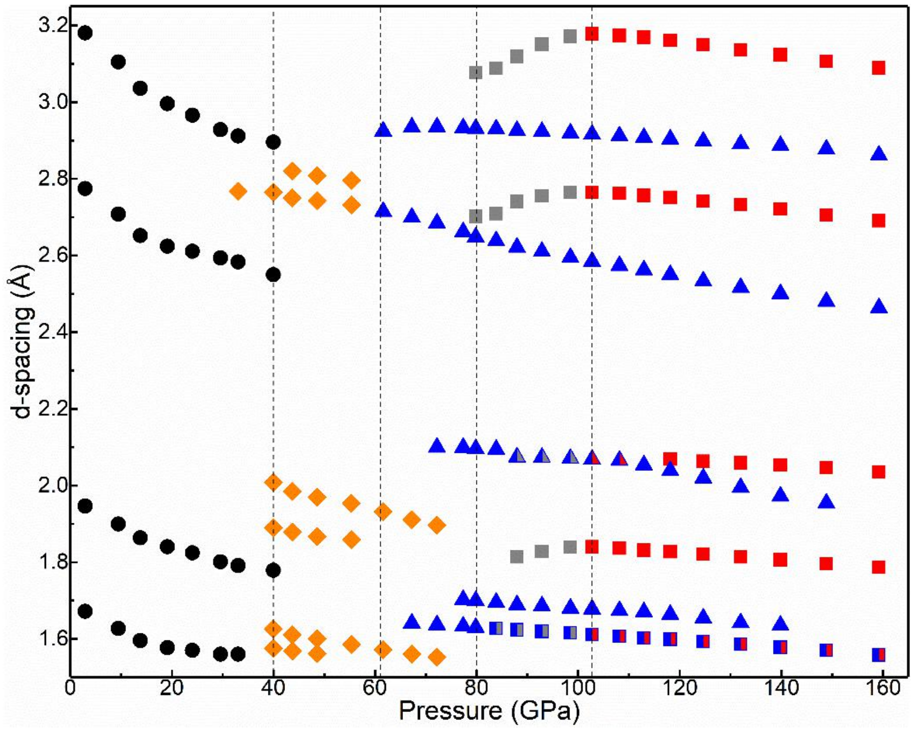

Supplementary Fig. 2 | Evolution of *d*-spacing of Ce polyhydrides with pressure. The vertical dashed lines indicate the phase transitions in Ce polyhydrides.

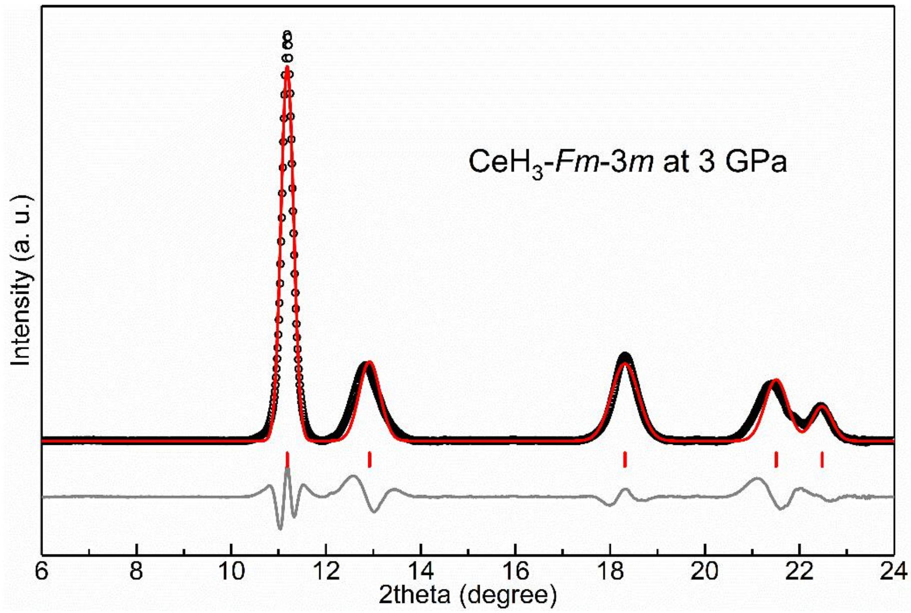

Supplementary Fig. 3 | The Rietveld refinement on CeH_3-*Fm*-3*m* at 3 GPa. The R factors are R_{wp} = 18.58 % and R_p= 12.68 % for the refinement.

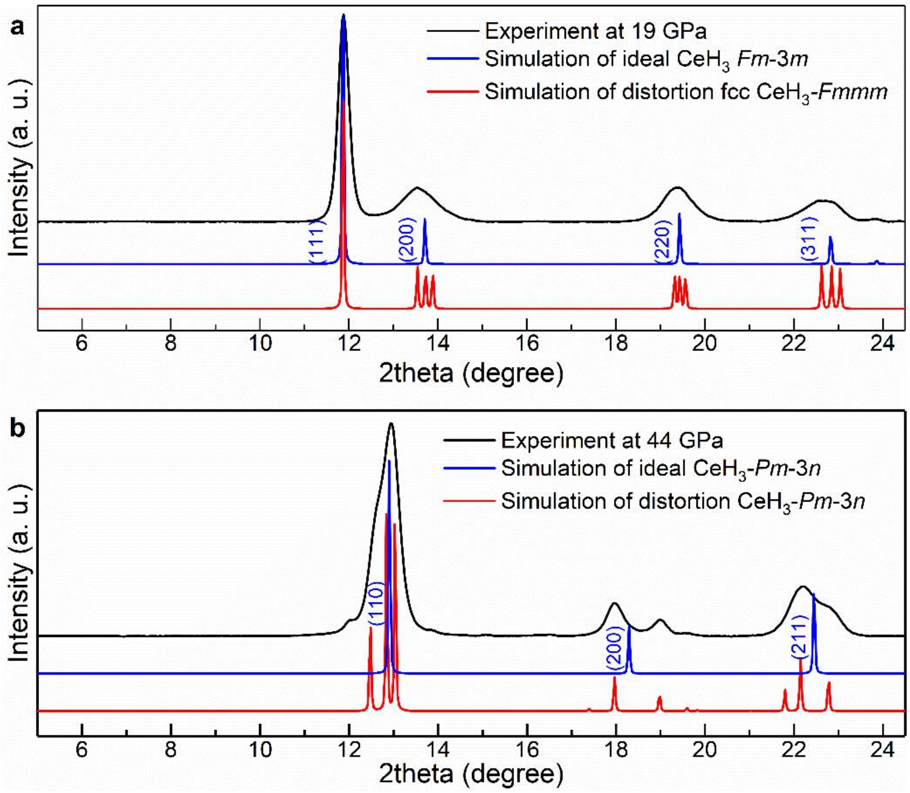

Supplementary Fig. 4 | Distortion of Ce sublattice at 19 GPa and 44 GPa. a, Comparation between ideal and distorted lattice of CeH_3-*Fm*-3*m* at 19 GPa. The diffraction peaks (200),(220),(311) have obviously anomalous broaden and shift, indicating a distortion in the fcc Ce sublattice. **b**, Comparation between ideal and distorted CeH_3-*Pm*-3*n* at 44 GPa.

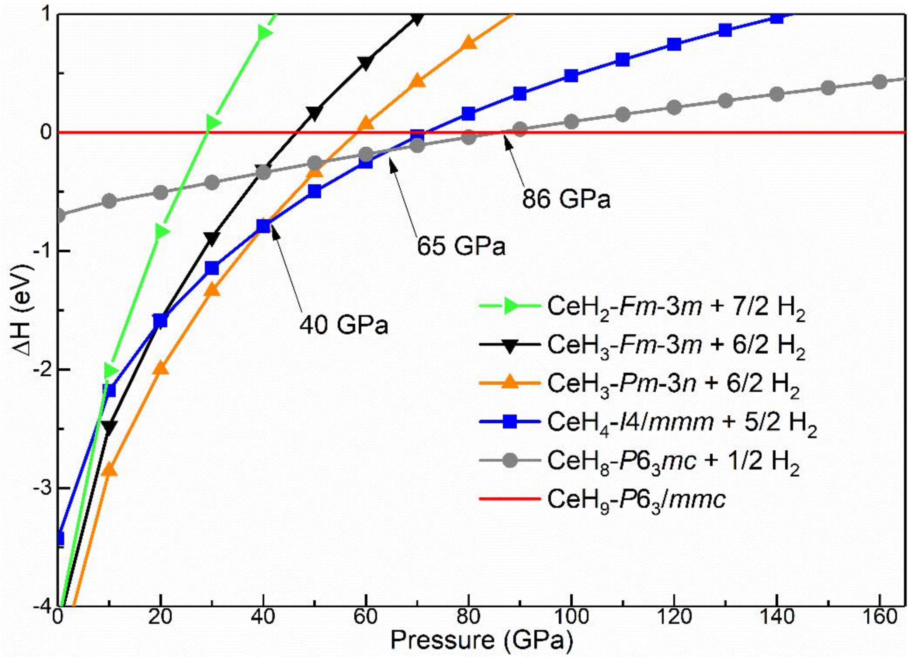

Supplementary Fig. 5 | The enthalpy of Ce polyhydrides as a function of pressure. $\Delta H = H(CeH_n)+(9-n)/2H(H_2)-H(CeH_9)$. The enthalpy of CeH_9 is used as the reference energy.

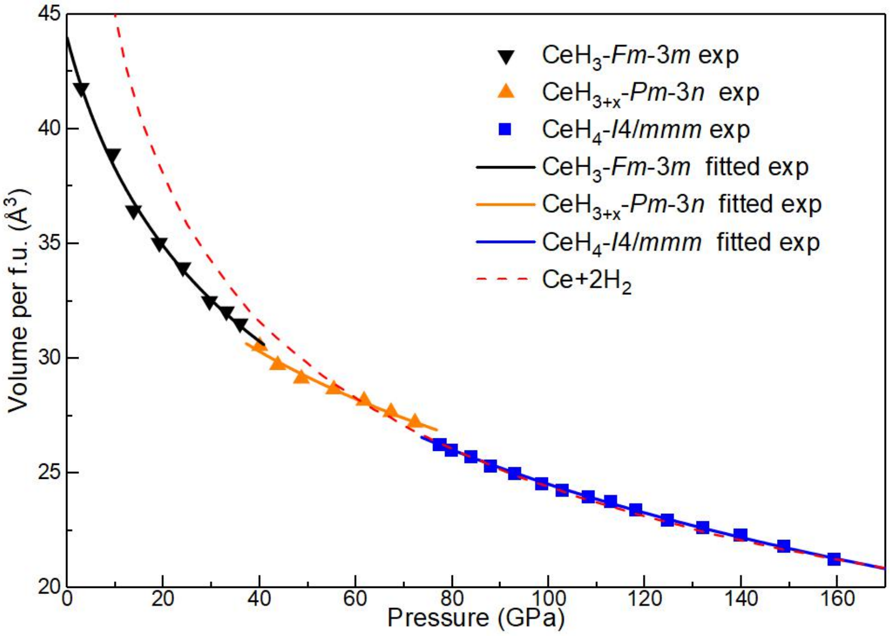

Supplementary Fig. 6 | The experimental volume of CeH_3, CeH_{3+x} and CeH_4 at different pressures.

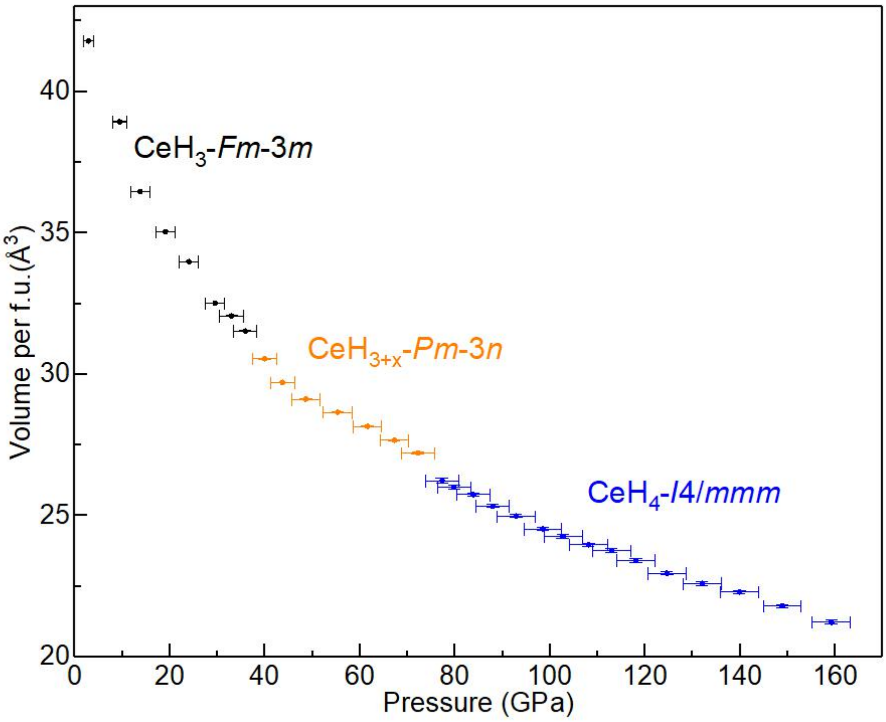

Supplementary Fig. 7 | The experimental volume of CeH_3, CeH_{3+x} and CeH_4 with error bars.

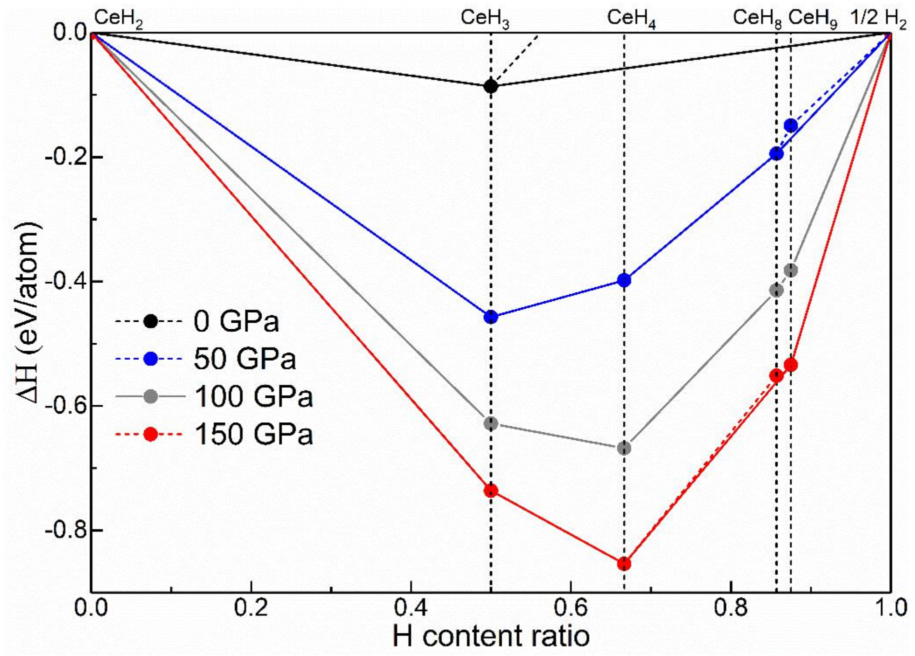

Supplementary Fig. 8 | The convex hull diagram of Ce polyhydrides at selected pressure. The structures of CeH_2-*Fm*-3*m*, CeH_3-*Pm*-3*n*, CeH_4-*I*4/*mmm*, CeH_8-*P*6$_3$*mc*, CeH_9-*P*6$_3$/*mmc* and H_2-*P*6$_3$/*m* and H_2-*C*2/*c* are considered respectively.

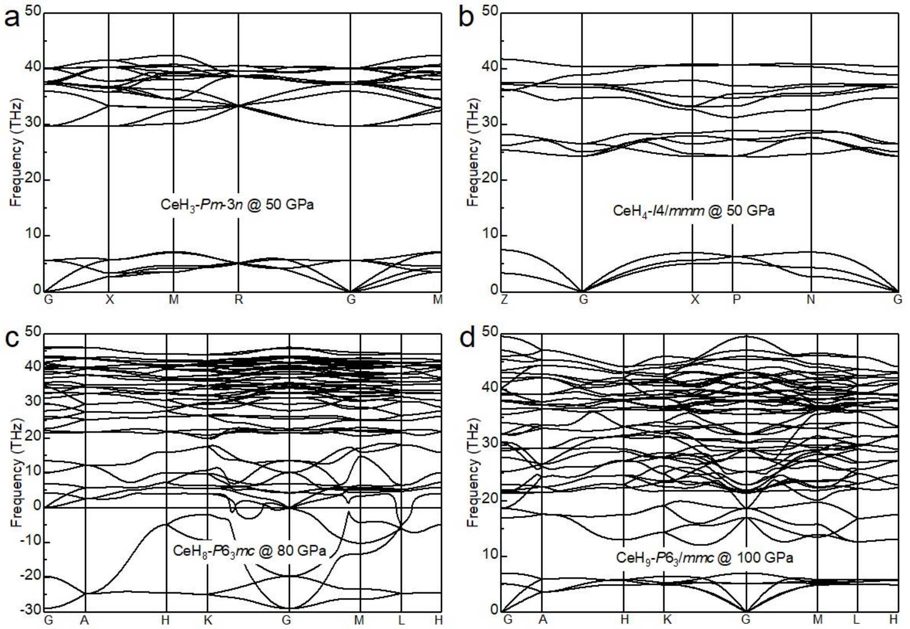

Supplementary Fig. 9 | (a-d) Phonon dispersion curves for CeH_3-*Pm*-3*n*, CeH_4-*I*4/*mmm*, CeH_8-*P*6$_3$*mc* and CeH_9-*P*6$_3$/*mmc* at 50, 50, 80 and 100 GPa, respectively. Imaginary phonons observed in CeH_8-*P*6$_3$*mc*.

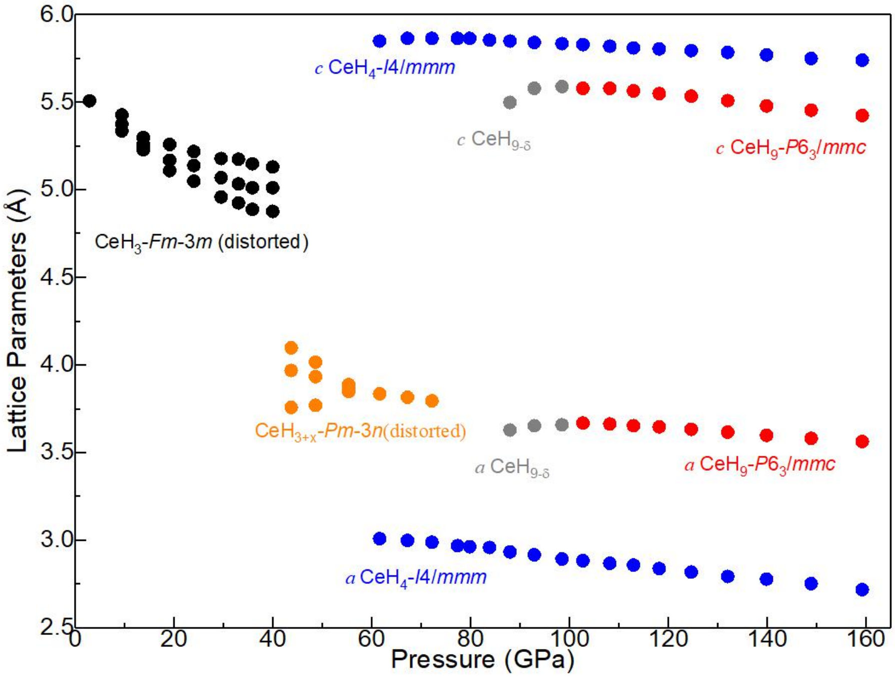

Supplementary Fig. 10 | Lattice parameters of Ce polyhydrides as a function of pressure. The evolution of lattice parameters of CeH_3-*Fm*-3*m*, CeH_{3+x}, CeH_4-*I*4/*mmm*, $CeH_{9-\delta}$, and CeH_9-*P*6_3/*mmc*.

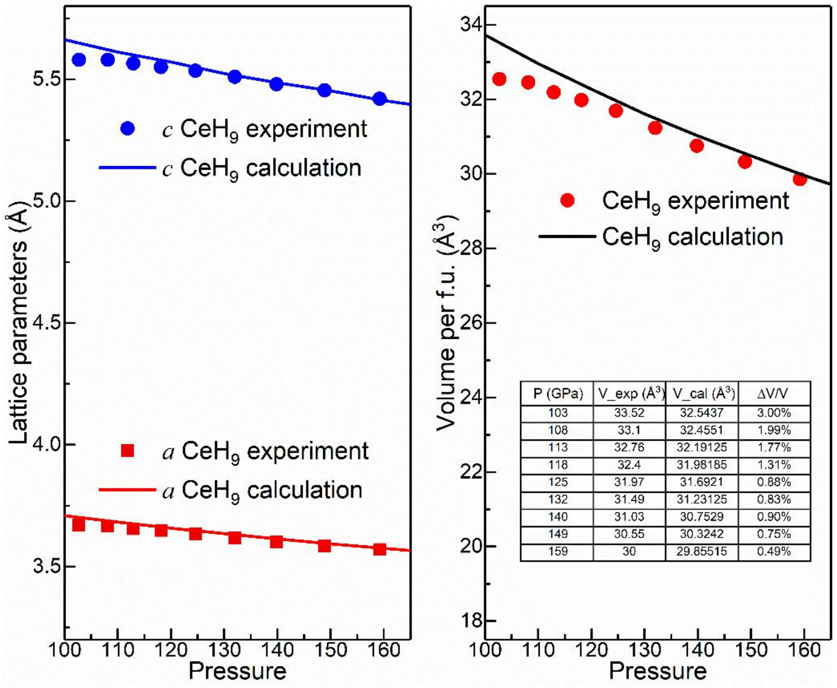

P (GPa)	V_exp ($Å^3$)	V_cal ($Å^3$)	ΔV/V
103	33.52	32.5437	3.00%
108	33.1	32.4551	1.99%
113	32.76	32.19125	1.77%
118	32.4	31.98185	1.31%
125	31.97	31.6921	0.88%
132	31.49	31.23125	0.83%
140	31.03	30.7529	0.90%
149	30.55	30.3242	0.75%
159	30	29.85515	0.49%

Supplementary Fig. 11 | Experimental and calculated lattice parameters and volume of CeH_9 at different pressures. a, The comparison between experimental and theoretical lattice parameters of CeH_9 at pressures, and b, The comparison between experimental and theoretical volume of CeH_9 at pressures. Inset is the volume difference under different pressures.

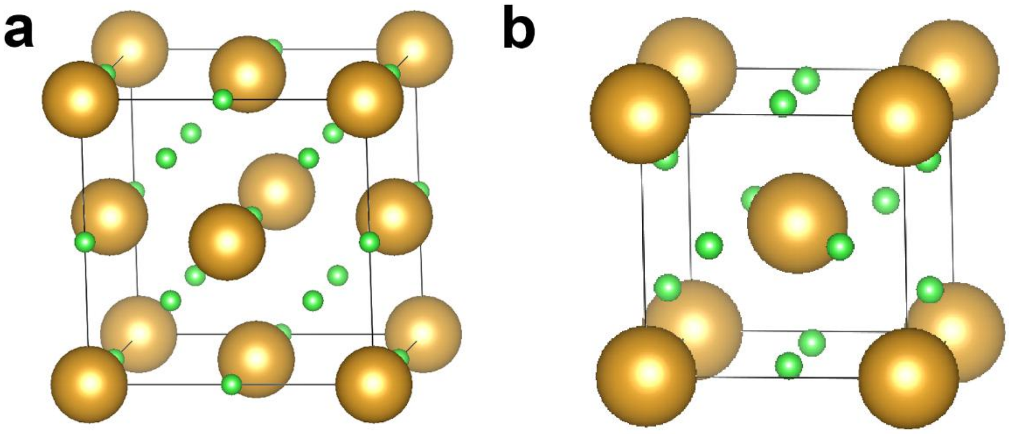

Supplementary Fig. 12 | The crystal structures of Ce polyhydrides. a, The crystal structure of CeH_3-*Fm*-3*m*. **b,** The crystal structure of CeH_3-*Pm*-3*n*.

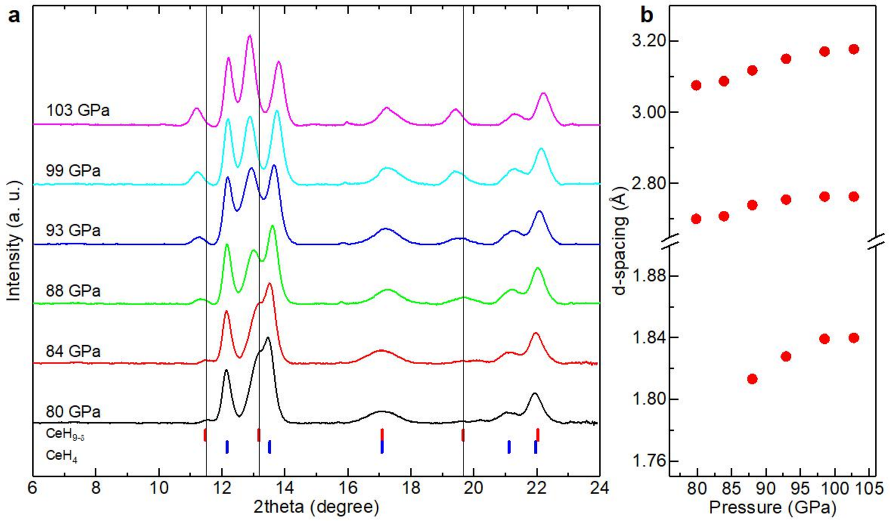

Supplementary Fig. 13 | The XRD patterns and evolution *d*-spacing of $CeH_{9-\delta}$. a, The XRD patterns in the pressure range 80-103 GPa. At the bottom, the patterns are contributed from $CeH_{9-\delta}$ and CeH_4-*I*4/*mmm*. With increasing pressure, the pattern shows the abnormal shift to lower angles, indicating the expansion of the volume and new phase with higher stoichiometry. b, Evolution of *d*-spacing of $CeH_{9-\delta}$ with pressure.

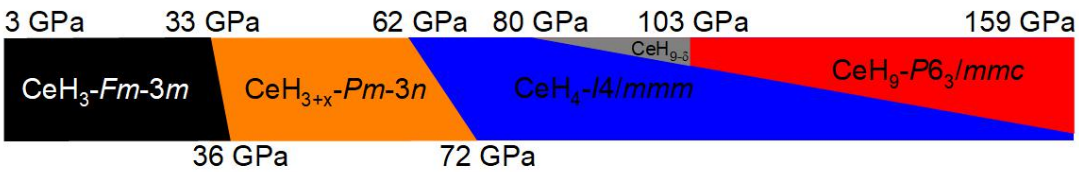

Supplementary Fig. 14 | Experimental phase diagram of Ce polyhydrides. The stable regions were confirmed via our experimental XRD patterns. The stoichiometry of Ce hydrides would increase with pressure.

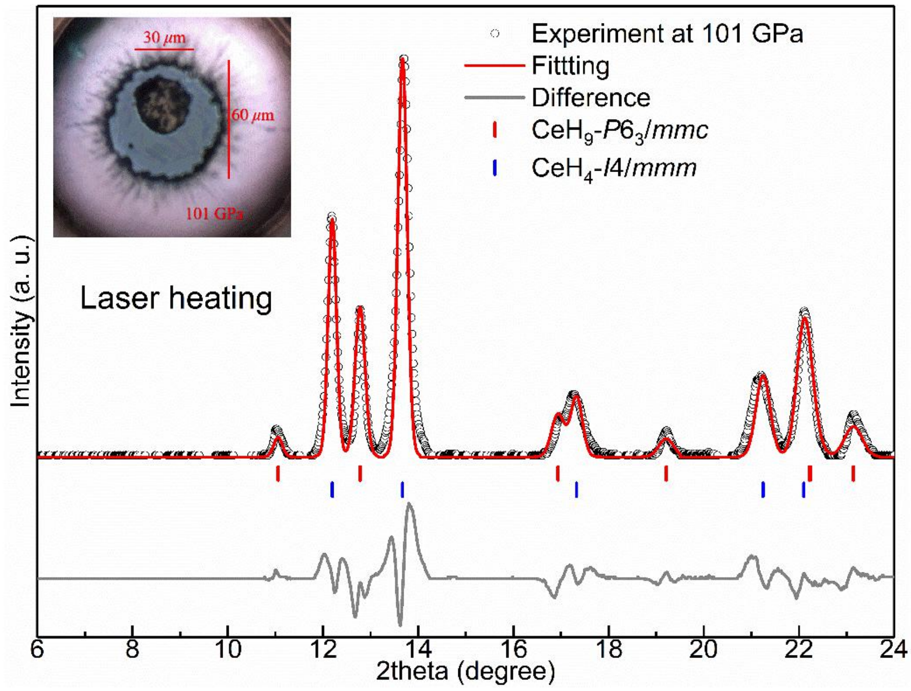

Supplementary Fig. 15 | The Rietveld refinements on new Ce polyhydrides at 101 GPa after laser-heating. The R factors are R_{wp} = 19.28 % and R_p= 13.04 % for the refinement.

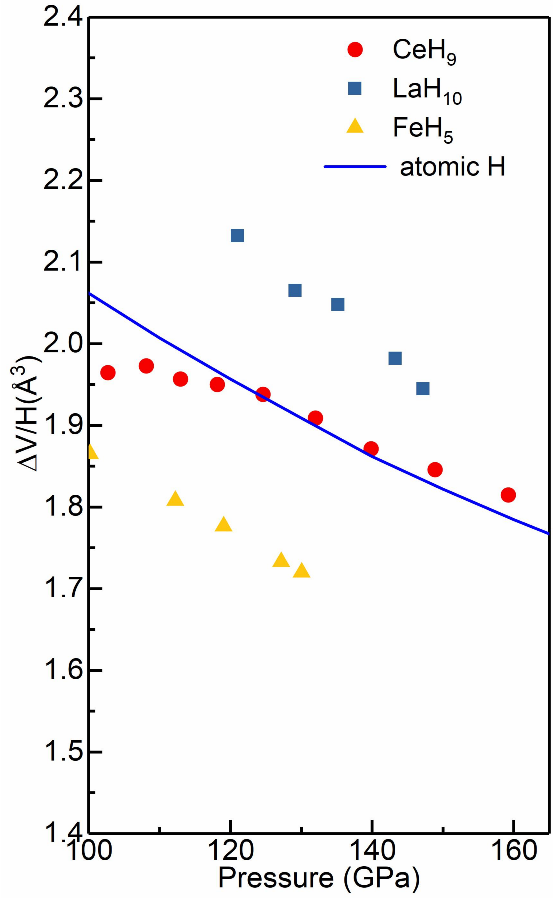

Supplementary Fig. 16 | The volume expansion per H atom.

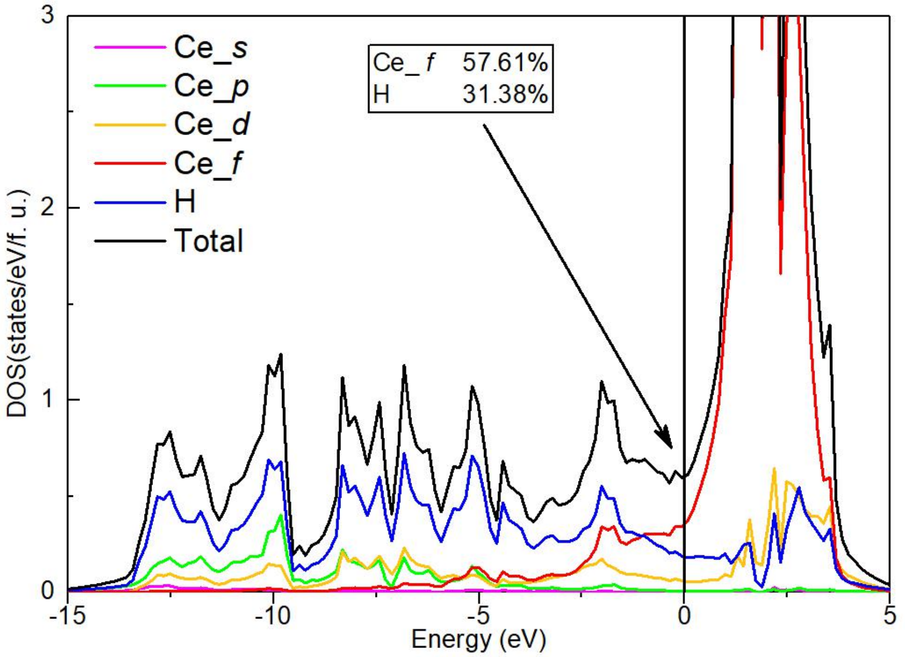

Supplementary Fig. 17 | The partial density of electronic states of CeH_9 at 100 GPa. The substantial contribution of Ce *f* electrons and H *s* electrons to the DOS at the Fermi level of CeH_9 with 31.38% by H and 57.61% by Ce 4*f* states.

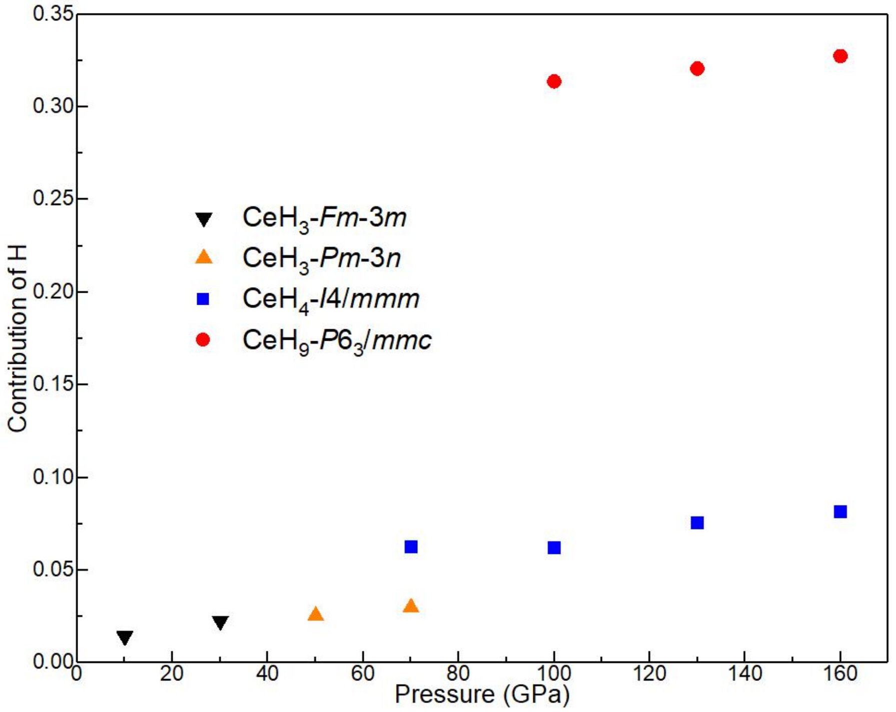

Supplementary Fig. 18 | The contribution of Hydrogen to the density of states at Fermi level in each phase.

Supplementary Table 1. Refined lattice parameters of Ce polyhydrides

	Pressure	Space group	Lattice Parameters	Atoms	Atomic coordinates		
					x	y	z
CeH_3	3 GPa	*Fm*-3*m*	*a*=5.509 Å	Ce(4*a*)	0	0	0
				H(4*b*)	1/2	0	0
				H(8*c*)	1/4	1/4	1/4
CeH_3	57 GPa	*Pm*-3*n*	*a*=3.811 Å	Ce(2a)	0	0	0
				H(6d)	0	1/4	1/2
CeH_4	76 GPa	*I*4/*mmm*	*a*=2.970 Å<br>*b*=5.865 Å	Ce(2*a*)	0	0	0
				H(4*d*)	0	1/2	1/4
				H(4*e*)	0	0	0.365
CeH_9	159 GPa	*P*6₃/*mmc*	*a*=3.565 Å<br>*b*=5.510 Å	Ce(2*d*)	2/3	1/3	1/4
				H(2*b*)	0	0	1/4
				H(4*f*)	1/3	2/3	0.149
				H(12*k*)	0.156	0.312	0.062
